# Functional roles of CD26/DPP4 in bleomycin‐induced pulmonary fibrosis

**DOI:** 10.14814/phy2.15645

**Published:** 2023-03-22

**Authors:** Yu Koyanagi, Takeshi Kawasaki, Yoshitoshi Kasuya, Ryo Hatano, Shun Sato, Yukiko Takahashi, Kei Ohnuma, Chikao Morimoto, Steven M. Dudek, Koichiro Tatsumi, Takuji Suzuki

**Affiliations:** ^1^ Department of Respirology, Graduate School of Medicine Chiba University Chiba Japan; ^2^ Department of Biomedical Science, Graduate School of Medicine Chiba University Chiba Japan; ^3^ Department of Therapy Development and Innovation for Immune Disorders and Cancers, Graduate School of Medicine Juntendo University Tokyo Japan; ^4^ Division of Pulmonary, Critical Care, Sleep and Allergy, Department of Medicine University of Illinois at Chicago Chicago Illinois USA; ^5^ Synergy Institute for Futuristic Mucosal Vaccine Research and Development Chiba University Chiba Japan

**Keywords:** CD26, dipeptidyl peptidase‐4, fibroblast, pulmonary fibrosis

## Abstract

The pathogenesis of pulmonary fibrosis involves complex interplay between cell types and signaling pathways. Recurrent alveolar epithelial injury can occur during pulmonary inflammation, causing dysregulation of epithelial repair. Dysregulated repair interacts with mesenchymal, inflammatory, and endothelial cells to trigger fibroblast‐to‐myofibroblast activation. CD26/dipeptidyl peptidase‐4 (DPP4) is a type II membrane protein mediating pleiotropic effect. However, the mechanistic role of CD26/DPP4 in pulmonary fibrosis remains unclear. In this study, we aimed to characterize *Dpp4* deficiency in a mouse bleomycin (BLM)‐induced pulmonary fibrosis model and in cell culture systems of human lung fibroblasts (HLFs). *Dpp4* knockout (*Dpp4* KO) mouse lungs exhibited lower Ashcroft scale indices, collagen content, and numbers of fibroblasts and myofibroblasts compared with those in C57BL/6 wild‐type (WT) mice. Upregulation of *Tgfb1* and *Tgfb2* mRNA levels in the lungs after BLM treatment was lower in *Dpp4* KO mice compared with those in WT mice. Although TGF‐β‐driven endothelial‐to‐mesenchymal transition (EndMT) has been implicated as one of the mechanisms of pulmonary fibrosis, a number of partial EndMT cells in lungs did not differ between *Dpp4* KO mice and WT mice. The proliferation capacity and mRNA levels of *COL1A1*, a collagen deposition‐related gene, in cultured HLFs were suppressed in *DPP4* small interfering RNA‐treated cells. This study indicates that the genetic deficiency of *DPP4* has protective effects against BLM‐induced pulmonary fibrosis, partly through the reduction in TGF‐β expression and inhibition of fibroblast activation in the lung. Our study suggests that CD26/DPP4 inhibition is a potential therapeutic strategy for pulmonary fibrosis.

## Graphical abstract

This current study has advanced our understanding of the functional role of CD26/DPP4 by demonstrating that CD26/DPP4 deficiency attenuates BLM‐induced pulmonary fibrosis in mice through a reduction in TGF‐β expression and inhibition of lung fibroblast activation.

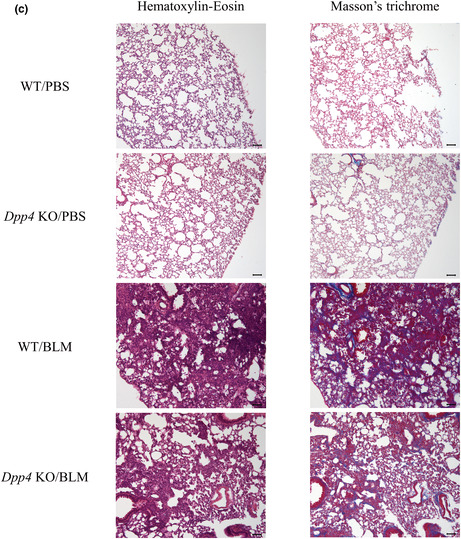

## 1 INTRODUCTION

Interstitial lung diseases (ILDs) comprise a large number of chronic progressive lung diseases that are characterized by varying degrees of inflammation, followed by pulmonary fibrosis in the lung interstitium. Although the clinical course is variable and unpredictable, these diseases can lead to progressive respiratory failure with poor prognosis (Raghu et al., 2006, 2011). The immune system plays a pivotal role in the initiation, development, and resolution of inflammation following recurrent alveolar epithelial injury and dysregulation of epithelial repair. The most accepted hypothesis for idiopathic pulmonary fibrosis (IPF) pathogenesis relies on the inability of alveolar epithelium to regenerate after injury. Further, IPF is characterized by altered composition and dysfunction of resident and immune cells in the lungs, leading to excessive accumulation of extracellular matrix (ECM) and progressive scarring. Although much progress has been made in understanding IPF pathogenesis and management, and disease‐modifying therapies have been approved worldwide, important clinical needs and demands are not yet met (Distler et al., 2019; Flaherty et al., 2019; Podolanczuk et al., 2021; Wollin et al., 2019).

The etiology of IPF is diverse and incompletely defined since all stages of fibrosis are accompanied by a broad spectrum of innate and adaptive immune responses. The pathogenesis of IPF involves complex interplay between cell types and signaling pathways. Repetitive alveolar epithelial cell injury may occur in the context of predisposing immunological inflammatory factors, leading to aberrant epithelial cell activation and dysregulation of epithelial repair (Heukels et al., 2019). Further, inflammation plays an important role in IPF pathogenesis (Heukels et al., 2019), although steroids as inflammatory modulators have exhibited deleterious effects in clinical IPF.

Repetitive alveolar epithelial injury triggers the early development of fibrosis. These injuries, in combination with dysregulated wound repair and fibroblast dysfunction, lead to tissue remodeling and pulmonary fibrosis, although tissue remodeling is complex and differs among compartments. Abnormal deposition of ECM proteins is a key factor in the development of tissue remodeling. The current notion is that myofibroblasts, which are derived from epithelial cells by epithelial–mesenchymal transition (EMT), exhibit abnormal proliferation and ECM overproduction, presumably resulting in IPF progression (Jia et al., 2021; Mackinnon et al., 2012; Podolanczuk et al., 2021; Salton et al., 2019; Wollin et al., 2019). When EMT occurs in the lung, E‐cadherin levels in epithelial cells decrease, and α‐smooth muscle actin (α‐SMA)‐expressing mesenchymal cells increase. A few studies have found that approximately one‐third of fibroblasts are of epithelial origin in pulmonary fibrosis. In addition, EMT plays a critical role in the development of pulmonary fibrosis (Della Latta et al., 2015; Jia et al., 2021; Nataraj et al., 2010; Sanchez‐Duffhues et al., 2018; Suzuki et al., 2017). The cytokine transforming growth factor β (TGF‐β) functions as an important mediator of fibrogenesis. Further, TGF‐β1 induces fibroblasts to undergo a phenotypic transition to myofibroblasts, which are effectors of the fibrotic state (Qian et al., 2018). Thus, the anti‐EMT pathway or inhibition of TGF‐β1 signaling is a novel potential target for IPF treatment.

The differentiation of fibroblasts into myofibroblasts favors the progression of fibrosis (Suzuki et al., 2020). Compared with fibroblasts, myofibroblasts upregulate α‐SMA expression and increase the production of ECM proteins (Akamatsu et al., 2013; Garrison et al., 2013; Hinz et al., 2007). Myofibroblasts can originate from sources other than fibroblasts. Endothelial cells are a potential source of endothelial‐to‐mesenchymal transition (EndMT) (Hashimoto et al., 2010). During this process, endothelial cells acquire a mesenchymal phenotype and present typical markers of myofibroblast differentiation, such as α‐SMA and vimentin, while reducing the expression of vascular endothelial cadherin (VE‐cadherin; Pardali et al., 2017). In one study, 16% of lung fibroblasts expressing α‐SMA and collagen type I were derived from lung endothelial cells in mice with bleomycin (BLM)‐induced pulmonary fibrosis (Hashimoto et al., 2010). Similar to EMT, TGF‐β plays a central role in promoting EndMT through a wide network of molecular interactions (Liu & Qi, 2020; Pardali et al., 2017).

CD26/dipeptidyl peptidase‐4 (DPP4) is a transmembrane protein expressed in various cells that exists as a soluble protein in tissue and circulation. The peptidase activity of CD26/DPP4 exerts effects on multiple proteins, including incretin hormones, and has led to the development of CD26/DPP4 inhibitors as therapeutic agents for diabetes. CD26/DPP4 also participates in immune regulation and promotion of inflammation (Morimoto & Schlossman, 1998; Ohnuma et al., 2008). In healthy human lung, CD26/DPP4 is expressed in type I and II alveolar epithelial cells, alveolar macrophages, vascular endothelium, and pleural mesothelium (Meyerholz et al., 2016).

We previously reported that CD26/DPP4 inhibition by the pharmaceutical agent sitagliptin ameliorated LPS‐induced lung injury in mice, with anti‐inflammatory effects on lung endothelial cells, and that CD26/DPP4 mediates inflammatory responses in the pulmonary endothelium (Kawasaki et al., 2018; Suzuki et al., 2016; Takahashi et al., 2021). These findings suggest plausible interactions between CD26/DPP4 and the pathophysiology of inflammatory lung diseases. Vildagliptin, another CD26/DPP4 inhibitor, ameliorates BLM‐induced pulmonary fibrosis (Liu & Qi, 2020), and *Dpp4* deficiency reduces BLM‐induced fibrosis in the skin and lungs of mice (Soare et al., 2020), suggesting a functional participation of CD26/DPP4 in pulmonary fibrosis. However, it remains unclear how CD26/DPP4 affects mechanisms related to pulmonary fibrosis, such as fibroblast activation or mesenchymal transition.

Mouse BLM‐induced pulmonary fibrosis is a widely used model of pulmonary fibrosis, because BLM damage provokes a histological lung pattern similar to that described in patients with IPF, and this pattern is characterized by patchy parenchymal inflammation, epithelial cell injury with reactive hyperplasia, EMT, activation and differentiation of fibroblasts to myofibroblasts, and basement membrane and alveolar epithelium injuries (Della Latta et al., 2015).

In this study, we aimed to clarify the functional roles of CD26/DPP4 in pulmonary fibrosis by focusing on fibroblast activation and EndMT using *Dpp4*‐deficient mice in the model of BLM‐induced pulmonary fibrosis (Liu et al., 2017), and in cultured human lung fibroblasts (HLFs).

## 2 MATERIALS AND METHODS

### 2.1 BLM‐induced pulmonary fibrosis in *Dpp4* knockout mice

Eight‐ 10‐week‐old male C57BL/6J wild‐type (WT) mice (Clea Japan) and *Dpp4* knockout (*Dpp4* KO) mice on a C57BL/6 background were intratracheally administered phosphate‐buffered saline (PBS) or 4 U/mg/body bleomycin (Nihon Kayaku). The lungs were harvested 21 days after BLM treatment. All animal experiments were conducted according to protocols approved by the Review Board for animal experiments of Chiba University, Japan.

### 2.2 Bronchoalveolar lavage fluid analysis

Bronchoalveolar lavage fluid (BALF) was collected 21 days after BLM treatment by instilling 1 mL of PBS through the tracheal cannula into the lungs, followed by slow recovery of the fluid. Cells were collected from the BALF by centrifugation (500 × g, 20 min, 4°C) and counted using an automated cell counter (TC20; Bio‐Rad). The BALF supernatant was centrifuged again (17,000 × g, 10 min, 4°C) and stored at −80°C until further analysis. The BALF protein concentration was measured using a bicinchoninic acid assay kit (Thermo Fisher Scientific).

### 2.3 Histological examination

Mouse lung tissues were fixed in formalin, embedded in paraffin, sectioned, mounted onto slides, and subjected to hematoxylin–eosin and Masson's trichrome staining. The severity of pulmonary fibrosis was semi‐quantitatively assessed according to the method proposed by Ashcroft (Ashcroft et al., 1988; Hubner et al., 2008).

### 2.4 Quantification of collagen content of lungs

The collagen content of lung tissues was measured using the Sircol Soluble Collagen Assay kit (Biocolor Ltd.) according to the manufacturer's instructions.

### 2.5 Preparation of single cell suspension from mouse lungs

At the time of harvest, the lungs were perfused from the right ventricle until blood‐free using 20 mL PBS containing 10 U/mL heparin (Mochida). They were then minced and digested in an enzyme cocktail of Dulbecco's Modified Eagle's Medium (Sigma) containing 1% bovine serum albumin (BSA) (Sigma), 2 mg/mL collagenase (Worthington), 100 μg/mL DNase (Sigma), and 2.5 mg Dispase II (Sigma) at 37°C for 60 min, and then meshed through a 70‐μm nylon cell strainer.

### 2.6 Flow cytometry analysis of mouse cells in BALF and lungs

Mouse cells in BALF or lungs were pretreated with anti‐CD16/32 antibody (BioLegend) for 10 min to block Fc receptors and then incubated with specific antibodies in the dark at 4°C for 15 min. The following antibodies were used for cell surface staining: anti‐CD31‐PE/Cy7 (BioLegend), anti‐CD45‐Alexa Flour 700 (BioLegend), anti‐CD326‐PerCP/Cyanine5.5 (BioLegend), and anti‐CD26‐FITC (BioLegend), and anti‐Gr‐1‐APC (BioLegend). After surface staining, the lung cells were incubated with α‐SMA (Thermo Fisher Scientific) and anti‐vimentin (Abcam) for 15 min in the dark at 4°C. The secondary antibody used was donkey anti‐rabbit IgG‐PE (Invitrogen) for 15 min in the dark at 4°C. Numbers of fibroblasts, myofibroblasts, and partial EndMT cells in the lungs were assessed using flow cytometry (FCM). Fibroblasts, myofibroblasts, and partial EndMT cells were defined as vimentin‐positive CD31^−^/CD45^−^/CD326^−^ cells (Lazarides, 1980; Morbini et al., 2011; Suzuki et al., 2017), α‐SMA‐positive CD31^−^/CD45^−^/CD326^−^ cells (Suzuki et al., 2017; Zhang et al., 2020), and α‐SMA‐positive CD31^+^/CD45^−^/CD326^−^ cells (Hashimoto et al., 2010; Suzuki et al., 2016, 2017), respectively. Cell fluorescence was measured using a BD FACS Canto™ II (BD Biosciences), and the data were analyzed using FlowJo software (TreeStar). To evaluate expression levels, mean fluorescence intensity (MFI) of a sample was calculated as follows: MFI of a sample stained with an antibody–MFI of a sample unstained (autofluorescence of the sample).

### 2.7 Culture of HLFs

Human lung fibroblasts were obtained from Lonza and cultured in Dulbecco's Modified Eagle's Medium, with L‐glutamine, phenol red, and sodium pyruvate, (FUJIFILM Wako) supplemented with 15% fetal bovine serum. The cells were incubated at 37°C in a 5% CO_2_ incubator and used at passages 6–8 for all experiments.

### 2.8 Transfections with small interfering RNA

Non‐specific control small interfering RNA (NS‐siRNA) (Cat# 4390843: Silencer™ Select Negative Control No. 1 siRNA), *DPP4* siRNA (Cat# 4392421: siRNA ID s4254) (*DPP4*‐siRNA1), and *DPP4* siRNA (Cat# 4392421: siRNA ID s4255) (*DPP4*‐siRNA2) were purchased from Thermo Fisher Scientific. For siRNA transfection, the Lipofectamine™ RNAiMAX Transfection Reagent (Invitrogen) was used according to the manufacturer's protocol. Cultured cells were transfected with siRNA at 60% confluence for 72 h. Selective silencing of CD26/DPP4 was confirmed using FCM and quantitative PCR (qPCR).

### 2.9 Real‐time quantitative PCR

Total RNA was extracted from cells using TRIzol, followed by the Direct‐zol RNA MiniPrep Plus Kit (Zymo Research Corporation). Subsequently, RNA was reverse‐transcribed via PCR using the SuperScript IV VILO Master Mix (Thermo Fisher Scientific) to synthesize single‐stranded cDNA. The cDNA samples were amplified using qPCR with Fast SYBR Green PCR Master Mix (Thermo Fisher Scientific) and the GeneAmp PCR System (Thermo Fisher Scientific). Specific primers were designed using the web software from the Universal Probe Library Assay Design Center (Roche Applied Science). The expression level of each target gene was normalized to hypoxanthine phosphoribosyltransferase 1 (HPRT1) threshold cycle (CT) values and calculated using the 2^−∆∆Ct^ method. ∆∆CT = (target gene CT of experimental group–reference gene CT of experimental group)–(target gene CT of control group–reference gene CT of control group).

### 2.10 Cell proliferation assay

Cell proliferation assay was performed to assess the number of viable cells using the Cell Counting Kit‐8 (WST‐8) (Dojindo Molecular Technologies) according to the manufacturer's protocol. HLFs (5000 cells/well) treated with siRNA against *DPP4* or non‐specific control siRNA for 72 h were detached using ACCUTASE (Thermo Fisher Scientific) and cultured in 96‐well plates for 24 h. The cells were then cultured with 10 μL of WST‐8 in each well at 37°C for 2 h. Cell viability was measured as the absorbance (optical density [OD]) at 450 nm using a microplate reader. The results were calculated using the following formula: Cell viability = (treatment group OD–blank group OD)/(control group OD–blank group OD).

### 2.11 Western blot analysis

Cells were washed with PBS, and lysates were prepared in Laemmli's SDS sample buffer (Boston Bioproducts). Protein samples were subjected to SDS‐PAGE and then transferred to polyvinylidene difluoride membranes (Thermo Fisher Scientific). After blocking with Tris‐buffered saline containing 0.1% Tween 20 (TBS‐T) and 5% BSA (Sigma) for 30 min at 25°C, the membrane was incubated with primary antibodies against α‐SMA (dilution 1:1000, Abcam) and β‐actin (dilution 1:1000, Thermo Fisher Scientific) for 1 h at 25°C. Secondary antibodies conjugated to horseradish peroxidase (dilution 1:2000, Thermo Fisher Scientific) were added to membranes for 30 min at 25°C. Finally, the SuperSignal West Dura kit (Thermo Fisher Scientific) was used to visualize the bands, and band densities were determined using the ImageJ software (National Institutes of Health).

### 2.12 Statistical analysis

Results were expressed as mean ± SD. One‐way ANOVA was used for multiple‐group comparisons, followed by Tukey's post hoc test. Student's t‐test was used to compare the two groups. Statistical analyses were performed using GraphPad Prism 5 software. Statistical significance was set at *p* < 0.05. To minimize distributions of values caused by possible differences in experimental conditions, data were normalized using the mean value derived from WT mice treated with BLM for each experiment, and then, the normalized values from each experiment were combined for statistical analysis.

## 3 RESULTS

### 3.1 BLM‐induced pulmonary fibrosis was attenuated in *Dpp4*‐deficient mice

**FIGURE 1:**
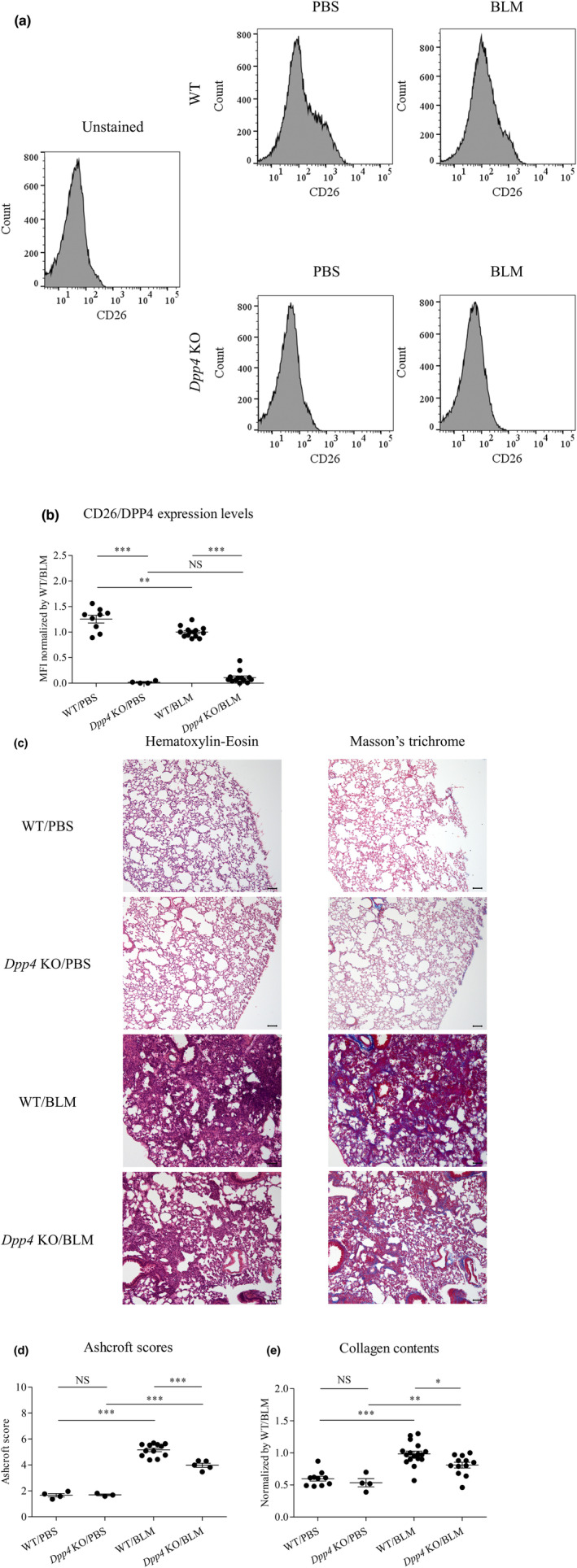
BLM‐induced pulmonary fibrosis in *Dpp4*‐deficient and WT mice. (a, b) CD26/DPP4 expression levels in lung cells of bleomycin (BLM)‐ or PBS‐treated *Dpp4* KO and WT mice were measured by mean fluorescence intensity (MFI) using flow cytometry analysis. CD26/DPP4 expression levels were substantially lower or nearly zero in *Dpp4* KO mice compared with those in WT mice (*n* = 4–19). (c) BLM‐induced pulmonary fibrosis was assessed using hematoxylin–eosin and Masson's trichrome staining in lung tissue sections in *Dpp4* KO and WT mice, respectively. Collagen fibers are differentially stained and shown as blue color in Masson's trichrome staining. Representative images are shown for each condition. Original magnification 100×. Black scale bar, 100 μm. (d and e) Fibrotic lung injury was histologically assessed by two independent researchers using the Ashcroft scoring system. Indices of Ashcroft scores and collagen content were lower in *Dpp4* KO mouse lungs than those in WT mouse lungs (n = 3–17). *p < 0.05, **p < 0.01, ***p < 0.001. Values represent the mean ± SD of three independent experiments. WT, wild‐type mice; NS, not significant.

CD26/DPP4 expression levels were evaluated in the cellular components of the mouse lung using FCM. CD26/DPP4 levels were confirmed to be substantially low or nearly zero in *Dpp4* KO mice, and the levels in WT mouse lung after BLM treatment were significantly lower than those after PBS treatment (Figure 1a,b). The effects of global *Dpp4* deficiency on BLM‐induced fibrosis were then assessed. Both Ashcroft scores and collagen content were significantly lower in *Dpp4* KO mouse lungs compared with those in WT mouse lungs (Figure 1c–e), suggesting that *Dpp4* deficiency has protective effects against BLM‐induced fibrosis in mice.

### 3.2 Severity of lung injury did not differ between *Dpp4*‐deficient mice and WT mice with BLM‐induced pulmonary fibrosis

**FIGURE 2:**
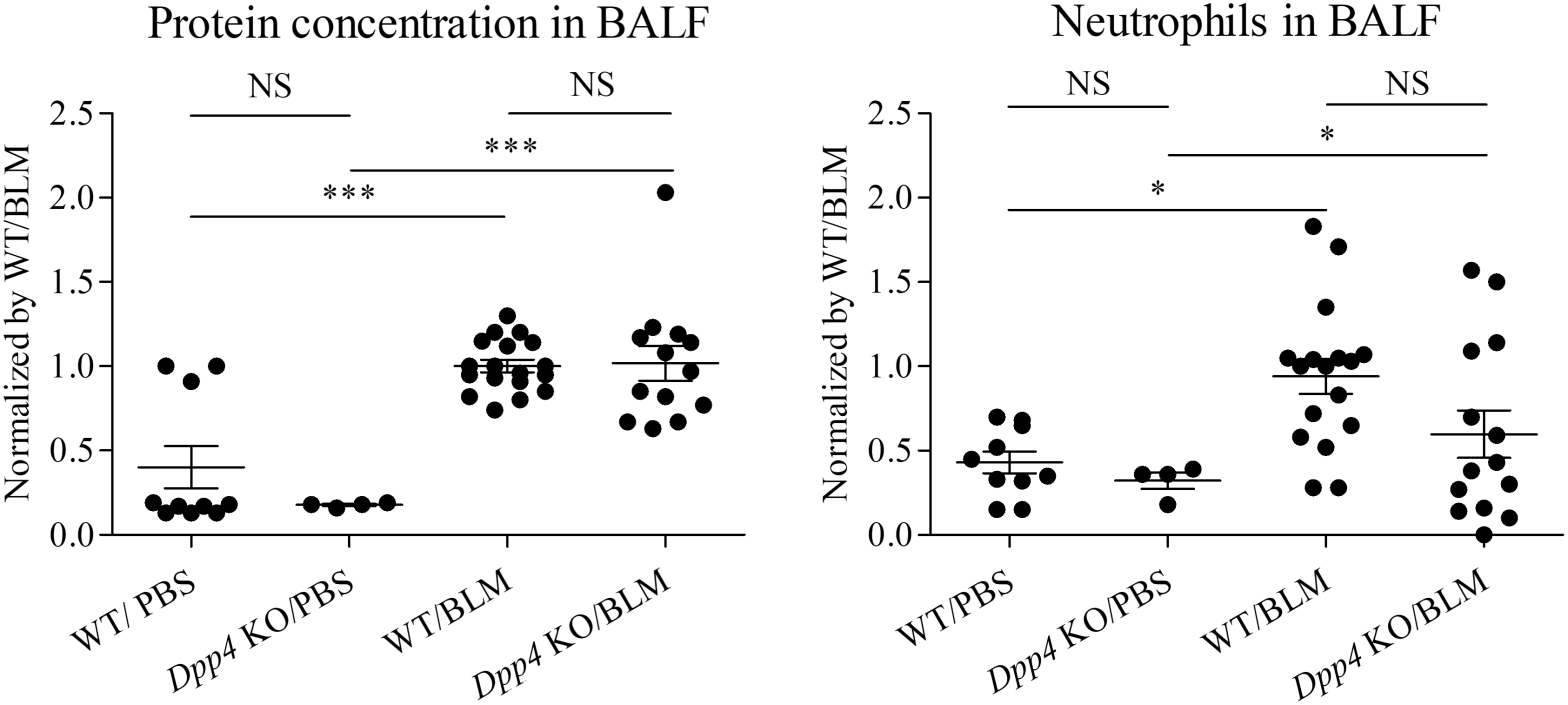
Severity of lung injury in *Dpp4*‐deficient and WT mice with BLM‐induced pulmonary fibrosis. Some representative lung injury parameters (total protein concentration and number of neutrophils) were measured in bronchoalveolar lavage fluid (BALF) of WT and *Dpp4* KO mice 21 days after bleomycin (BLM) or PBS treatment. Neither total protein concentration levels nor the number of neutrophils in BALF significantly differed between WT and *Dpp4* KO mice, in either the PBS or BLM‐treated groups (*n* = 4–18). *p < 0.05, ***p < 0.001. Values represent the mean ± SD of three independent experiments. WT, wild‐type mice; NS, not significant.

In pulmonary fibrosis, the immune system plays a pivotal role in the initiation, development, and resolution of parenchymal inflammation following an insult or damage to organs. The role of inflammation as an important component of IPF etiology is controversial and is sometimes seen as an epiphenomenon of fibrosis. We investigated whether severity of lung injury, as evaluated by some representative parameters of inflammation and permeability (Matute‐Bello et al., 2011), was ameliorated by *Dpp4* deficiency in BLM‐induced pulmonary fibrosis. The results revealed that protein concentrations and the number of CD45^+^ and Gr‐1 double positive neutrophils in BALF were increased by BLM treatment but did not differ between the *Dpp4* KO mice and WT mice (Figure 2). These results suggest that the severity of lung injury may not differ between *Dpp4*‐deficient mice and WT mice with BLM‐induced pulmonary fibrosis.

### 3.3 The numbers of lung fibroblasts and myofibroblasts in BLM‐induced lung fibrosis were lower in *Dpp4*‐deficient mice than in WT mice

**FIGURE 3:**
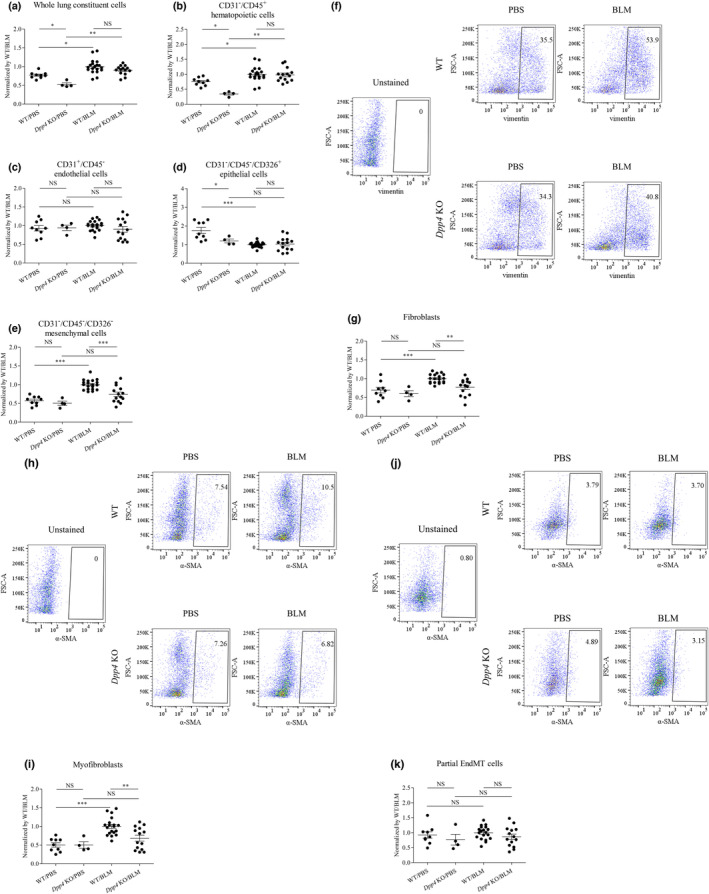
Lung constituent cells from *Dpp4‐*deficient and WT mice in BLM‐induced lung fibrosis. Numbers of mouse whole lung cells and lung constituent cells were evaluated using flow cytometry (FCM) analysis (*n* = 4–19). (a) The number of whole lung constituent cells in WT mice was increased after BLM challenge but did not differ between WT mice and *Dpp4* KO mice. (b) Similarly, the number of CD31^−^/CD45^+^ hematopoietic cells was increased after BLM challenge but did not differ between WT mice and *Dpp4* KO mice with BLM‐induced pulmonary fibrosis. (c) Neither the number of CD31^+^/CD45^−^ endothelial cells in WT mice differ after BLM challenge, nor the number of CD31^+^/CD45^−^ endothelial cells differ between WT mice and *Dpp4* KO with BLM‐induced pulmonary fibrosis. (d) Meanwhile, the number of CD31^−^/CD45^−^/CD326^+^ epithelial cells in WT mice was decreased after BLM challenge, and the number did not differ between WT mice and *Dpp4* KO mice with BLM‐induced pulmonary fibrosis. (e) Of note, the number of CD31^−^/CD45^−^/CD326^−^ mesenchymal cells in WT mice was increased after BLM challenge, and the magnitude of this increase was significantly lower in *Dpp4* KO mice than that in WT mice. (f, g, h, and i) Additionally, the numbers of fibroblasts and myofibroblasts in *Dpp4* KO mouse lungs after BLM treatment were all lower than those in WT mice. (j and k) Meanwhile, the number of partial EndMT cells in lungs after BLM challenge did not differ between in *Dpp4* KO mice and in WT mice. (F, H, and J) Representative FCM panels are shown. These cell numbers are not normalized by lung size or body weight. *p < 0.05, **p < 0.01, ***p < 0.001. Values represent the mean ± SD of three independent experiments. WT, wild‐type mice; NS, not significant.

We next assessed numbers of the lung constituent cells using FCM analysis that were defined as follows: CD31^+^/CD45^−^ endothelial cells, CD31^−^/CD45^+^ hematopoietic cells, CD31^−^/CD45^−^/CD326^+^ epithelial cells, and CD31^−^/CD45^−^/CD326^−^ mesenchymal cells (Kawasaki et al., 2015). The number of whole lung constituent cells in WT mice was increased after BLM challenge but did not differ between WT mice and *Dpp4* KO mice (Figure 3a). Similarly, the number of CD31^−^/CD45^+^ hematopoietic cells was increased after BLM challenge but did not differ between WT mice and *Dpp4* KO mice with BLM‐induced pulmonary fibrosis (Figure 3b). Neither the number of CD31^+^/CD45^−^ endothelial cells in WT mice differ after BLM challenge, nor the number of CD31^+^/CD45^−^ endothelial cells differ between WT mice and *Dpp4* KO with BLM‐induced pulmonary fibrosis (Figure 3c). Meanwhile, the number of CD31^−^/CD45^−^/CD326^+^ epithelial cells in WT mice was decreased after BLM challenge, and the number did not differ between WT mice and *Dpp4* KO mice with BLM‐induced pulmonary fibrosis (Figure 3d). Of note, the number of CD31^−^/CD45^−^/CD326^−^ mesenchymal cells in WT mice was increased after BLM challenge, and the magnitude of this increase was significantly lower in *Dpp4* KO mice than that in WT mice (Figure 3e). Additionally, the numbers of fibroblasts and myofibroblasts in *Dpp4* KO mouse lungs after BLM treatment were all lower than those in WT mice (Figure 3f,g,h,i), while the number of partial EndMT cells in lungs after BLM challenge did not differ between in *Dpp4* KO mice and in WT mice (Figure 3j,k). These results suggested that lung fibroblast activation in the lungs were suppressed by *Dpp4* deficiency in BLM‐induced lung fibrosis.

### 3.4 Expression levels of CD26/DPP4 in the WT mouse lung after BLM‐challenge were lower in epithelial and endothelial cells, while higher in hematopoietic and mesenchymal cells

**FIGURE 4:**
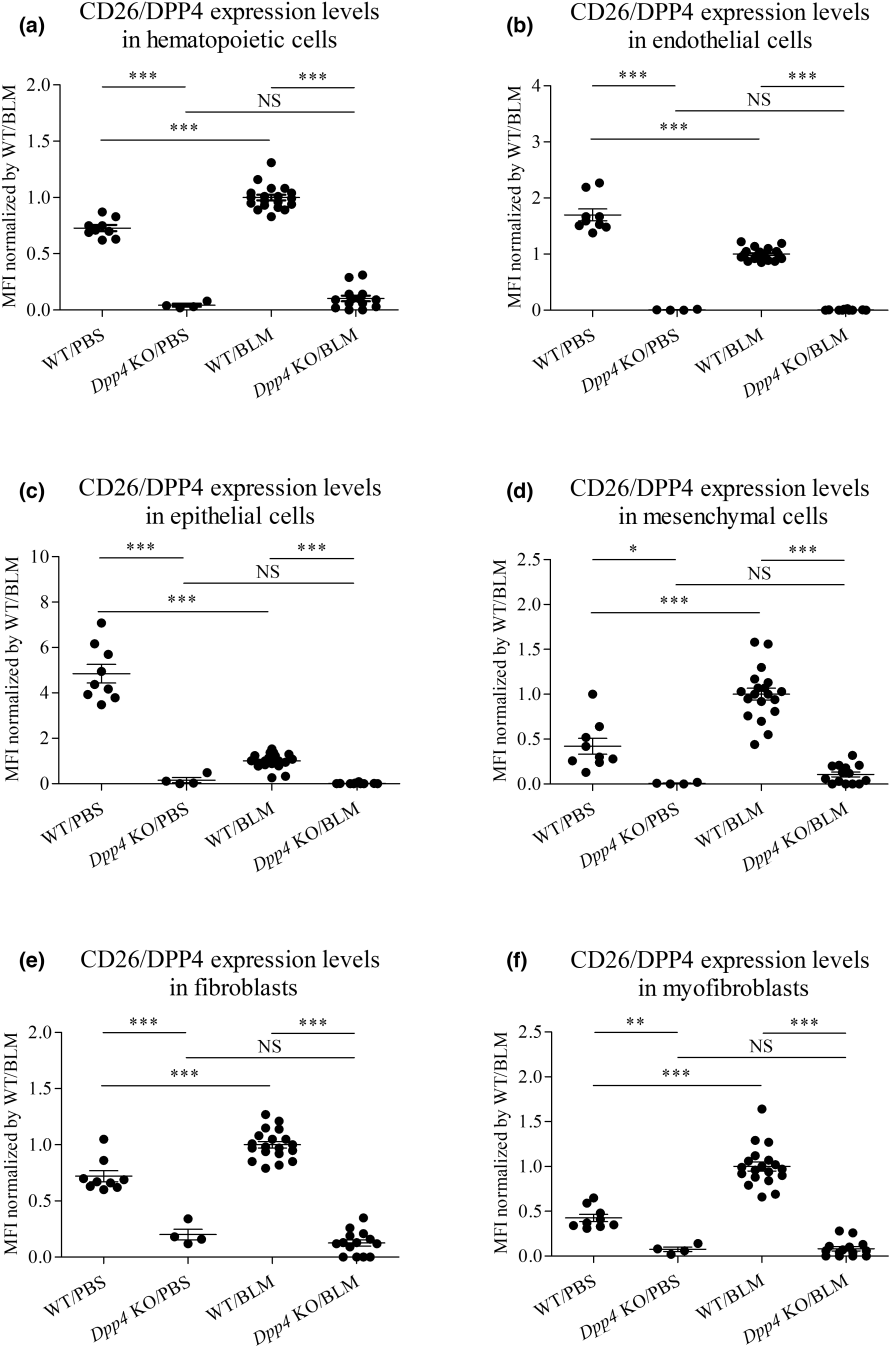
CD26/DPP4 expression levels in lung constituent cells from *Dpp4‐*deficient and WT mice in BLM‐induced lung fibrosis. CD26/DPP4 expression levels in lung constituent cells of bleomycin (BLM)‐ or PBS‐treated *Dpp4* KO and WT mice were measured by mean fluorescence intensity (MFI) using flow cytometry analysis: (a) CD31^−^/CD45^+^ hematopoietic cells, (b) CD31^+^/CD45^−^ endothelial cells, (c) CD31^−^/CD45^−^/CD326^+^ epithelial cells, (d) CD31^−^/CD45^−^/CD326^−^ mesenchymal cells, (e) vimentin‐positive CD31^−^/CD45^−^/CD326^−^ fibroblasts, and (f) α‐SMA‐positive CD31^−^/CD45^−^/CD326^−^ myofibroblasts. CD26/DPP4 expression levels were substantially lower or nearly zero in *Dpp4* KO mice compared with those in WT mice regardless of BLM treatment (*n* = 4–19). *p < 0.05, **p < 0.01, ***p < 0.001. Values represent the mean ± SD of three independent experiments. WT, wild‐type mice; NS, not significant.

As shown in Figure 1b, CD26/DPP4 levels in WT mouse lung after BLM treatment were significantly lower than those after PBS treatment. Therefore, we next determined CD26/DPP4 levels in lung constituent cells using FCM analysis. The results demonstrated that lung expression levels of CD26/DPP4 after BLM‐challenge were lower in epithelial and endothelial cells (Figure 4b,c), while they were higher in hematopoietic and mesenchymal cells, including fibroblasts and myofibroblasts (Figure 4a,d,e,f), compared with those after PBS treatment. These results suggest that the decreased expression levels of CD26/DPP4 in WT mouse lungs after BLM treatment may be due to effects in epithelial and endothelial cells. Additionally, CD26/DPP4 levels in the lung constituent cells were all confirmed to be substantially low or nearly zero in *Dpp4* KO mice regardless of BLM treatment (Figure 4a–f).

### 3.5 Expression levels of *Tgfb* in *Dpp4*‐deficient mouse lungs were lower than those in WT mice after BLM treatment

**FIGURE 5:**
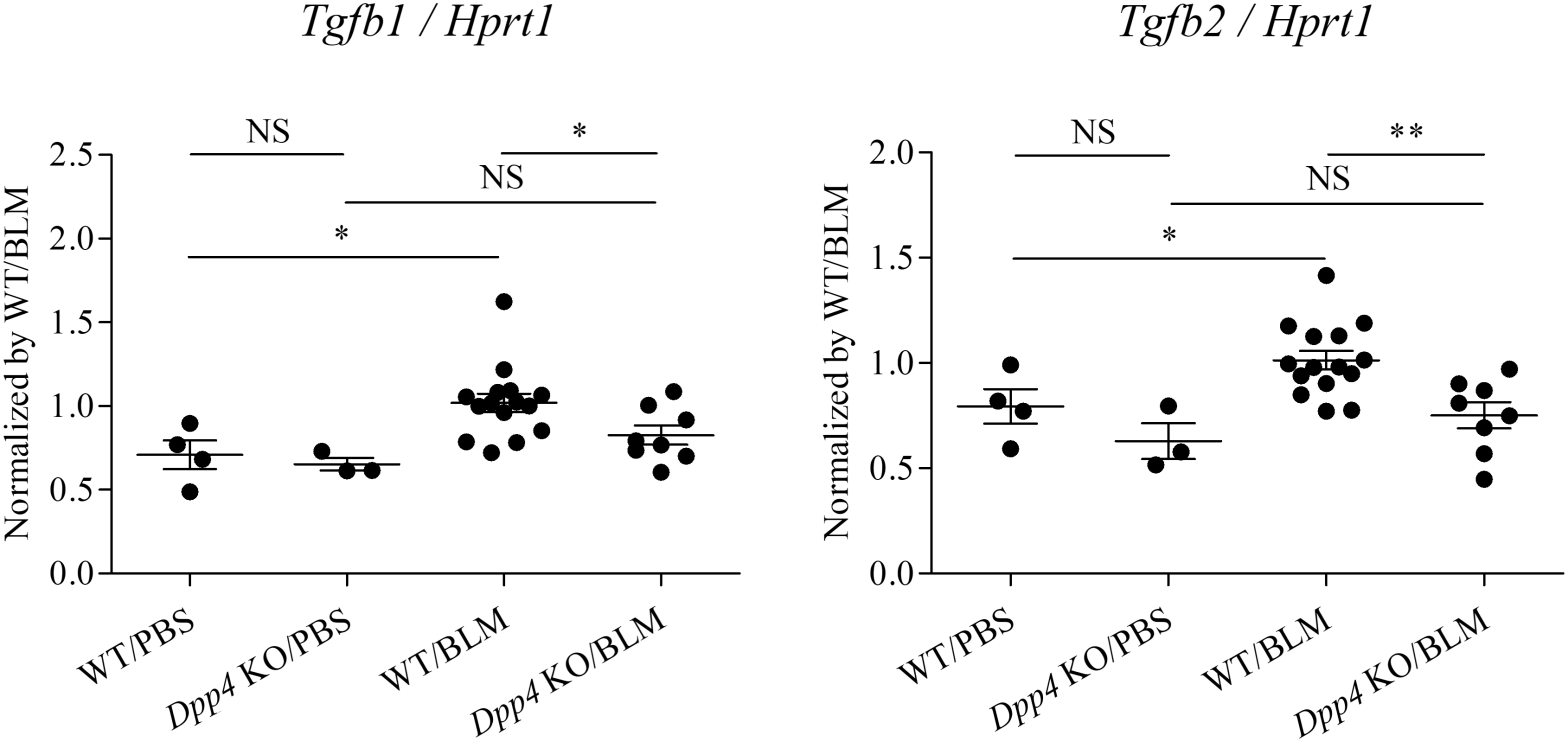
*Tgfb* expression levels in *Dpp4*‐deficient and WT mice in BLM‐induced lung fibrosis. *Tgfb1* and *Tgfb2* mRNA expression levels in lung cells were compared between WT and *Dpp4* KO mice relative to hypoxanthine phosphoribosyltransferase (*Hprt*) as reference values for internal standardization of real‐time PCR data. The mRNA levels of *Tgfb1* and *Tgfb2* were higher in the WT lungs after bleomycin (BLM) treatment, respectively. Both were lower in the lungs of *Dpp4* KO mice after BLM exposure (*n* = 3–15). *p < 0.05, **p < 0.01. Values represent the mean ± SD of three independent experiments. WT, wild‐type mice; NS, not significant.

We next assessed the mRNA expression levels of the central pro‐fibrotic genes, *Tgfb1* and *Tgfb2*, in the lungs. The levels of both *Tgfb1* and *Tgfb2* were upregulated in WT mouse lungs after BLM treatment, while those in *Dpp4*‐deficient mice were significantly lower than those in WT mice (Figure 5). These results suggest that upregulation of the central fibrotic genes, *Tgfb1* and *Tgfb2*, was suppressed by *Dpp4* deficiency in BLM‐induced lung fibrosis.

### 3.6 Cell proliferation capacity and collagen‐related gene expression of HLFs were suppressed by *DPP4* siRNA knockdown

**FIGURE 6:**
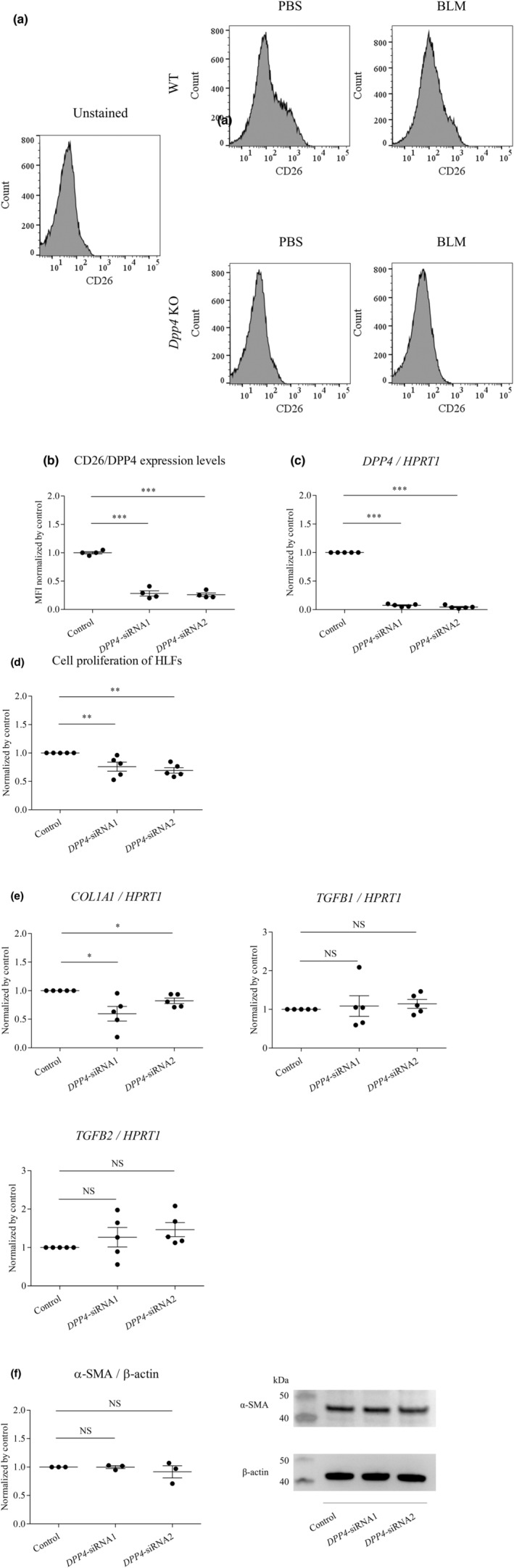
Cell proliferation capacity and pro‐fibrotic gene expression of HLFs after siRNA‐mediated *DPP4* knockdown. (a and b) Protein levels of CD26/DPP4 expression in cultured human lung fibroblasts (HLFs) were lower in siRNA1 and siRNA2 treatment compared with the treatment by non‐specific control RNA measured by mean fluorescence intensity (MFI) using flow cytometry analysis (*n* = 4). (c) *DPP4* mRNA expression levels in cultured HLFs were measured using quantitative PCR relative to hypoxanthine phosphoribosyltransferase (*HPRT*) as a reference value after siRNA1 and siRNA2 treatments. mRNA levels were lower in HLFs treated with *DPP4*‐siRNA1 and siRNA2 (*n* = 5). (d) The proliferation of human lung fibroblasts (HLFs) was determined in the WST‐8 assay. The WST‐8 assay results suggested that knockdown of *DPP4* decreased the viability of HLFs (*n* = 5). (e) mRNA expression levels of *TGFB1*, *TGFB2*, and *COL1A1*, relative to those of the internal control *HPRT*, in human lung fibroblasts (HLFs) after *DPP4*‐siRNA treatment were measured using real‐time quantitative PCR. mRNA levels of *COL1A1* were downregulated in HLFs by siRNA treatment. mRNA levels of *TGFB1* and *TGFB2* were not downregulated in HLFs by siRNA treatment (*n* = 5). (f) Protein levels of α‐SMA were not significantly suppressed in HLFs after siRNA treatment (*n* = 3). *p < 0.05, **p < 0.01, ***p < 0.001. Values represent the mean ± SD of three independent experiments. NS, not significant; Control, non‐specific control siRNA.

Lung fibroblast activation is considered to be a major mechanism of pulmonary fibrosis (Suzuki et al., 2020; Wynn & Ramalingam, 2012), and data in this current study indicated that the number of lung fibroblasts was lower in *Dpp4* deficient mice with BLM‐induced pulmonary fibrosis than that in WT mice (Figure 3g). Therefore, we next assessed the functional role of CD26/DPP4 in lung fibroblasts using cultured HLFs. After 72 h of siRNA treatment, CD26/DPP4 expression in cultured HLFs was suppressed at the protein (Figure 6a,b) and mRNA levels (Figure 6c) compared with those in HLFs treated with non‐specific siRNA. The effects of *DPP4* suppression in HLFs by siRNA were evaluated in terms of the proliferative capacity of HLFs using the WST‐8 assay. The results revealed that the knockdown of *DPP4* inhibited the proliferation of HLFs (Figure 6d) We next examined whether expression levels of genes known as fibroblast activation markers, *TGFB1*, *TGFB2*, and *COL1A1*, were transcriptionally inhibited by *DPP4* siRNA treatment in HLFs in vitro. As a result, the mRNA levels of *COL1A1*, a collagen deposition‐related gene, were significantly downregulated in transfected HLFs transfected with either *DPP4*‐siRNA1 or siRNA2, whereas *TGFB1* and *TGFB2* were not downregulated (Figure 6e). The expression levels of α‐SMA, an activation marker of fibroblasts, were not significantly suppressed in HLFs transfected with either *DPP4*‐siRNA1 or siRNA2 (Figure 6f). These results suggested that inhibition of proliferation capacity and the downregulation of a collagen deposition‐related gene in HLFs by suppressing CD26/DPP4 expression could be associated with the attenuation of pulmonary fibrosis in vivo.

## 4 DISCUSSION

In this study, we demonstrated that in *Dpp4* KO mice BLM‐induced pulmonary fibrosis was significantly attenuated and the numbers of fibroblasts and myofibroblasts in the lungs were lower compared to those in WT mice. Additionally, expression levels of *Tgfb1* and *Tgfb2* in the lungs were lower in *Dpp4*‐deficient mice than those in WT mice, and suppression of CD26/DPP4 expression by siRNA treatment had inhibitory effects on lung fibroblast activation in vitro. Meanwhile, *Dpp4* deficiency was unlikely to be related to EndMT regulation in mouse BLM‐induced pulmonary fibrosis. Our current study not only confirmed that BLM‐induced pulmonary fibrosis is attenuated in *Dpp4* KO mice (Soare et al., 2020), but it expands upon prior work by suggesting that the mechanisms responsible for these effects in vivo involve a decrease in fibroblasts and myofibroblasts, *Tgfb* downregulation in the lungs, and inhibitory effects on lung fibroblast activation.

Fibroblasts in pulmonary fibrosis acquire an active myofibroblast phenotype, which is thought to be a major mechanism underlying the development of pulmonary fibrosis (Suzuki et al., 2020; Wynn & Ramalingam, 2012). Although the molecular mechanisms underlying the persistent activation of fibroblasts remain incompletely understood, TGF‐β signaling has emerged as a core pathway for fibroblast activation in pulmonary fibrosis. In our study, the downregulation of the expression levels of *Tgfb1* and *Tgfb2* in the lungs was demonstrated, suggesting a central mechanism to ameliorate BLM‐induced pulmonary fibrosis. Previous studies reported that under proinflammatory conditions, TGF‐β is produced by immune cells, epithelial cells (Juban et al., 2018), or platelets (Meyer et al., 2012) in a latent form. Therefore, *DPP4* deficiency in those cell types might relate to the low expression levels of *Tgfb1* and *Tgfb2* in the lungs with BLM‐induced pulmonary fibrosis. The substrates of CD26/DPP4 include a broad range of mediators, and CD26/DPP4 modulates intracellular signaling through direct interactions with regulatory molecules. Further, CD26/DPP4 is expressed in tissue‐resident cells such as fibroblasts (but not in *Dpp4* KO mice) and may regulate fibroblast activation and collagen release in a TGF‐β‐dependent manner. Of note, our study demonstrated that CD26/DPP4 expression levels in lung mesenchymal cells, including fibroblasts and myofibroblasts, were increased in BLM‐induced fibrosis (Figure 4d–f). One study demonstrated that TGF‐β drives the expansion of CD26/DPP4‐positive fibroblasts and the upregulation of CD26/DPP4 promotes the activation of TGF‐β pathways in fibroblasts (Soare et al., 2020). CD26/DPP4 inhibition reduces the TGF‐β‐induced activation of extracellular signal‐regulated kinase (ERK) signaling in cultured human fibroblasts, and ERK inhibition ameliorates experimental fibrosis. However, other intracellular cascades than ERK are also regulated by TGF‐β signaling, and some of these are not affected by CD26/DPP4 inhibition (Soare et al., 2020). One review paper mentions that the inhibition of CD26/DPP4 may regulate TGF‐β production in a disease‐specific manner mainly depending on the cell type that are involved. CD26/DPP4 inhibition has no major effects on systemic TGF‐β levels; however, it generally results in a local reduction of TGF‐β expression in fibrotic disease (Ohm et al., 2022). Taken together with the results in the current study, these studies suggest a close link among CD26/DPP4, regulation of TGF‐β expression, and lung fibroblast activation.

The present study also demonstrated that both proliferative capacity and a collagen‐related gene expression were suppressed in HLFs with its CD26/DPP4 expression reduced by *DPP4*‐siRNA treatment in vitro (Figure 6d,f). These results suggests that the reduction in CD26/DPP4 expression inhibits fibroblast activation. Previous studies reported that CD26/DPP4 expression is locally regulated in fibrotic tissues and CD26/DPP4 is a marker of activated skin fibroblasts (Soare et al., 2020), while its expression may not be associated with an activated fibroblast phenotype in human lung fibroblasts from IPF patients (Kadefors et al., 2022). Together, fibroblasts can be a heterogeneous population, even in the same lung tissue, and CD26/DPP4 expression is a marker of activation in some fibroblasts. The possible mechanisms how CD26/DPP4 expression and proliferative capacity and a collagen‐related gene expression are related remains unclarified; therefore, further research is warranted.

Although the exact etiology of pulmonary fibrosis is unknown and likely diverse, all stages of fibrosis are accompanied by innate and adaptive immune responses. The role of inflammation as an important component in the etiology of pulmonary fibrosis has been controversial. BLM administration develops lung inflammation in the early stage of the BLM‐induced mouse model. In this process, the expression of *Tgfb1* and *Smad3* increased, followed by the activation of JAK–STAT pathways, while the knockdown of IL‐31 decreased STAT1 expression (Shi et al., 2014). In the present study, lung injury parameters of protein concentrations and the number of neutrophils in BALF 21 days after BLM treatment demonstrated no significant differences between *Dpp4* KO and WT mice (Figure 2), suggesting that neutrophilic inflammation is not associated with the amelioration of fibrosis in *Dpp4* deficiency in the fibrotic stage of BLM‐induced lung fibrosis. However, previous studies demonstrated some immunomodulatory effects of CD26/DPP4 as follows. CD26/DPP4 possesses non‐enzymatic functions, acting as a T‐cell costimulatory protein and interacting with ECM proteins such as collagen and fibronectin (Itou et al., 2013; Ohnuma et al., 2008). The T‐cell costimulatory activity of CD26/DPP4 affects the inflammatory process in pulmonary fibrosis, although its target immune cells or pulmonary constitutive cells have not been elucidated (Hatano et al., 2015). Additionally, the beneficial effects of pharmacological CD26/DPP4 inhibition on ischemia–reperfusion and LPS‐induced lung injuries through its anti‐inflammatory effects on pulmonary endothelial cells have been previously reported (Jungraithmayr et al., 2012). Taken together, it might be possible in our current study that anti‐inflammatory effects of *Dpp4* deficiency on BLM‐induced lung inflammation at the early stage could lead to the amelioration of pulmonary fibrosis at the later stage.

Our current observations are limited in several aspects. First, the source of the downregulation of the central fibrotic expression of *Tgfb1* and *Tgfb2* in mouse BLM‐induced fibrosing lungs has not been identified. The global *Dpp4* KO mice used in this study do not allow for cell‐specific determination of genetic deficiency. Our in vitro study (Figure 6e) indicates that other cell types than fibroblasts could be candidate cell types and should be examined. Second, our study demonstrated that the effects of BLM on CD26/DPP4 expression levels were different among various cell types in mouse lung during BLM‐induced fibrosis (Figures 4a–f). However, the key mechanisms associated with CD26/DPP4 signaling that are involved in the pathophysiology of pulmonary fibrosis are still unclear. Although downregulation of *Tgfb* in lungs of BLM‐induced pulmonary fibrosis is considered to be an important mechanism (Figure 5), TGF‐β1/Smad signaling pathway includes both positive and negative regulatory ones in each constituent cell such as fibroblasts, endothelial cells, and EndMT cells. TGF‐β1 activates the PKB, JNK, and AKT signaling pathways through the PI3K (Wang et al., 2022). Other signaling pathways including Wnt/β signaling pathway through miR‐133 could be involved in the pathogenesis of pulmonary fibrosis (Wei et al., 2019). Third, it remains unknown whether decreased expression of TGF‐β1 and TGF‐β2 is a correlative or causative finding. Experiments with overexpression or gain of TGF‐β function would be a helpful future direction to determine whether TGF‐β expression level is a causative mechanism. Fourth, investigation using human lung specimens or data mining from published databases would be helpful to characterize the potential importance of CD26/DPP4 in fibrosis during human disease. Lastly, it would be helpful to clarify what substrates of CD26/DPP4 are involved in these effects, and to examine the effects of CD26/DPP4 activation on fibroblasts or other cell types. Further studies are warranted to better understand the functional role of CD26/DPP4 in pulmonary fibrosis, since multiple important questions that need to be addressed remain unanswered.

In conclusion, our study demonstrated that genetic deficiency of *Dpp4* has protective effects on BLM‐induced pulmonary fibrosis in mice through a reduction in *Tgfb* expression levels in the lungs and direct inhibitory effects on lung fibroblast activation. These results suggest that CD26/DPP4 inhibition is a potential therapeutic strategy for pulmonary fibrosis.

## AUTHOR CONTRIBUTIONS

T.K. and Y.K.—Conceptualization; Y.K. and T.K.—Investigation; T.K., K.T., and T.S.—Funding acquisition; T.K., R.H., Y.T., S.S., and K.O.—Methodology; T.K.—Project administration; Y.K., C.M., K.T., and T.S.—Supervision; Y.T. and T.K.—Writing‐original draft; Y.K., R.H., Y.T., S.S., K.O., C.M., S.M.D., K.T., and T.S.—Writing‐review and editing.

## FUNDING INFORMATION

This research was funded by JSPS KAKENHI (grant numbers 19K17663, 22K16163, and 22H03076), AMED‐CREST (JP21gm1210003), AMED (223fa627003h0001), and a research grant from the Intractable Respiratory Diseases and Pulmonary Hypertension Research Group, Ministry of Health, Labor and Welfare, Japan (grant numbers 20FC1027 and 21FC1027).

## CONFLICT OF INTEREST STATEMENT

The authors declare no conflict of interest.

## ETHICS STATEMENT

All animal experiments were conducted according to protocols approved by the Review Board for animal experiments of Chiba University, Japan.
